# Awareness of Antibiotic Resistance Among Medical Professionals in Kerala, India: A Cross-Sectional Study

**DOI:** 10.7759/cureus.95204

**Published:** 2025-10-23

**Authors:** Abdul Shameem Kuttikkunnummal, Sarinkumar P Sivan, Deepa Divakaran, Muhamed Salahudeen, Pooja Vijay, Huda Junaina Theruvinkattil, Shurooq A Usman

**Affiliations:** 1 Acute Medicine, Janardan Hospital, Kasargod, IND; 2 Community Medicine, Kannur Medical College Anjarakandy, Kannur, IND; 3 Microbiology, Kannur Medical College Anjarakandy, Kannur, IND; 4 Emergency Medicine, Orchid Hospital Changaramkulam, Malappuram, IND; 5 Neurosurgery, Sree Gokulam Medical College, Thiruvananthapuram, IND; 6 Internal Medicine, Travancore Medical College, Kollam, IND; 7 Medicine, College of Basic Medical Sciences, Jilin University, Changchun City, CHN

**Keywords:** amr awareness, antibiotic prescription, antimicrobial resistance, antimicrobial resistance (amr), antimicrobial stewardship programs

## Abstract

Background: Antimicrobial resistance (AMR) has emerged as a critical global health threat, undermining the effective treatment of infectious diseases. The growing challenge of antibiotic resistance is a matter of concern, driven by frequent misuse of antimicrobial agents and limited awareness among healthcare professionals. This study aimed to assess the level of antibiotic resistance awareness across diverse medical professionals in Kerala, India, and to identify demographic and contextual influences that affect awareness and stewardship practices.

Methods: A cross-sectional survey was conducted among 345 healthcare professionals, including doctors, dentists, nurses, and pharmacists across Kerala. Participants completed a validated 23-item Antibiotic Resistance Awareness Scale, supplemented with modules on demographics, formal training, and workplace factors. Data were analyzed to evaluate predictors of awareness scores.

Results: Participants demonstrated moderate to high overall AMR awareness (mean score 73.9 ± 10.5), with the highest scores among physicians (75.8) and the lowest among nurses (69.7, p < 0.001). Significant disparities were observed by profession, training level, and practice setting. Only 23.5% reported formal AMR training, with pharmacists showing the highest training participation (44%). Males scored higher than females (mean difference 2.3, p=0.046), and practitioners in tertiary care had superior knowledge compared to those in primary care (mean difference 3.4, p=0.008). Training participation showed a positive but nonsignificant association with awareness (p=0.074).

Conclusions: While overall awareness of antibiotic resistance among Kerala’s healthcare professionals was good, substantial disparities exist, especially among nurses and those lacking formal AMR training. These findings underscore the urgent need for targeted educational programs, integrated stewardship curricula, and resource strengthening. Addressing these gaps is essential for successful AMR mitigation and for informing future policy and curricular reforms locally and in similar contexts globally.

## Introduction

Antimicrobial resistance (AMR) has become one of the most urgent global health threats of the 21st century, threatening the effectiveness of antibiotics and undermining decades of progress in infectious disease management [1]. AMR manifests when microorganisms undergo evolutionary processes leading to their resistance against antimicrobial medications commonly employed for treating such infections [2]. Factors contributing to the spread include inappropriate prescription practices, inadequate patient education, limited diagnostic facilities, unauthorized sale of antimicrobials, lack of appropriate functioning drug regulatory mechanisms, and non-human use of antimicrobials, such as in animal production [3]. Alarmingly, resistant infections are a leading cause of death around the world, with the highest burdens in low-resource settings. In developing countries, the absence of reliable surveillance systems and the inadequate dissemination of research findings often leave healthcare professionals without timely and accurate information on AMR patterns within their populations [3].

Global Research on Antimicrobial Resistance (GRAM) project findings for 2019 show that an estimated 4.95 million people died with a drug-resistant bacterial infection, and that more than 1.27 million of those deaths are directly attributable to AMR. Projections suggest that these deaths can rise to 10 million deaths per year by 2050, surpassing the combined global mortality rates of cancer and road traffic accidents [4]. The World Health Organization warns that if unchecked, AMR will make even common infections untreatable and devastate health systems worldwide. According to economic projections, antimicrobial resistance could lead to a 2-3.5% decline in global GDP by 2050, along with a 3-8% reduction in livestock production, potentially costing the global economy up to USD 100 trillion [5].

In India, the situation is particularly alarming due to various factors like unrestricted access to antibiotics, inadequate infection prevention and control measures, and the absence of stringent guidelines to mitigate the emergence of AMR. Studies highlighted the need for a coordinated, multifaceted strategy to combat AMR in India [6]. India has proactively responded to the global threat of antimicrobial resistance by adopting the National Action Plan on Antimicrobial Resistance (NAP-AMR) in 2017, which is in alignment with the World Health Organization’s Global Action Plan on AMR. This comprehensive framework emphasizes a One Health approach, integrating human, animal, and environmental health sectors to tackle AMR through multifaceted strategies. The NAP-AMR outlines six strategic priorities for tackling AMR in India. The main objective of Strategic Priority 1 is to improve awareness and understanding of AMR through effective communication, education, and training. It proposes to revise the professional curriculum of all sectors and industries related to AMR, such as human, animal, environmental, pharmaceutical, and food, following a One Health approach [7]. 

Over the past two decades, Kerala has implemented decentralization in public governance, a reform widely recognized as one of the most significant institutional innovations globally. In 2018, Kerala became the first state in India to launch a sub-national action plan on antimicrobial resistance, developed within a One Health framework to guide future action on antimicrobial resistance [8]. Kerala, despite having a relatively advanced healthcare system compared to other Indian states, continues to experience a high level of AMR. Local studies have highlighted gaps in awareness and practical application among healthcare professionals as well as the lack of implementation of effective antimicrobial stewardship programs [9]. Healthcare workers are key to controlling AMR through rational prescribing and effective stewardship. Yet, recent studies underscore significant gaps in awareness and education regarding AMR, especially among nurses and pharmacists. Many professionals lack formal AMR training, contributing to suboptimal antibiotic use. Despite national and global action plans, the success of interventions hinges on the knowledge and practices of frontline healthcare providers [6].

This study aimed to assess awareness of antimicrobial resistance among major healthcare professions in Kerala (doctors, nurses, pharmacists, and dentists) using a validated 23-item scale. It compared awareness scores across different professional groups, training levels, and practice settings. Additionally, the study examined the relationship between attendance in AMR training and awareness levels. 

## Materials and methods

Study design

A cross-sectional survey was conducted among healthcare professionals practicing in Kerala, India. 

Participants and sampling 

The study targeted licensed healthcare professionals working in Kerala from the following groups: doctors, dentists, nurses, and pharmacists. Participants were invited to join the study through a convenience sampling method. Survey invitations were distributed via professional networks, hospital mailing lists, and social media. Healthcare workers who declined to participate or submitted incomplete responses were excluded from the study.

Sample size calculation 

Sample size was calculated using the formula n = (Z2 × p(1 - p)) / d2. Assuming a 95% confidence level (Z = 1.96), an expected proportion of 50% (p = 0.5), and a 5% margin of error (d = 0.05), the estimated sample size is approximately 384. To account for potential non-responses and incomplete questionnaires, a target of 450 respondents was set [10].

Data collection instrument 

Antibiotic resistance awareness among healthcare professionals was assessed using the validated 23-item scale developed by Pinto Jimenez et al. (2023). This scale was specifically created for use in low- and middle-income countries. This instrument is free to use for non-commercial research under a Creative Commons Attribution-Non-Commercial License (CC BY-NC 4.0). As per the license, non-commercial reuse, distribution, and reproduction in any medium are permitted, provided the original work is properly cited [11]. 

The instrument includes four modules: 1) Demographics: Information on country, profession, gender, age, level of training, type of care, and practice setting. 2) ABR Training: Items evaluating formal training on antibiotic resistance and continuing education experiences. 3) ABR Awareness Scale: A 23-item module assessing knowledge on the mechanisms, drivers, transmission, and control of antibiotic resistance, with responses recorded on a four-point Likert scale. 4) Contextual Factors: Items addressing workplace factors that may influence antibiotic prescription and dispensing. 

Data collection procedure 

The questionnaire was deployed using the Kobo Collect platform, an open-source tool for digital data collection. KoboToolbox and KoboCollect are free to use for non-commercial academic and humanitarian research under the terms of the GNU Lesser General Public License v3.0 (LGPL-3.0), which allows use, modification, and distribution provided the license is respected [12]. A digital survey link was shared with potential participants via email and social media. Informed consent was obtained at the beginning of the survey, ensuring that participation was voluntary and responses were anonymized. 

Data management and analysis 

Data exported from Kobo Collect were analyzed using R software version 4.5.1 [12,13]. Descriptive statistics summarized participants’ demographic and practice characteristics and AMR awareness scores. Internal consistency of the 23-item AMR Awareness Scale was evaluated with Cronbach’s alpha [11]. Group comparisons of mean awareness scores across professions and other categorical variables were performed using one-way ANOVA and independent t-tests as appropriate. Associations between categorical demographic variables and profession were assessed using chi-square tests. Correlations between continuous variables were examined with Pearson’s correlation coefficient. Statistical significance was set at p < 0.05.

## Results

Participant characteristics

A total of 345 healthcare professionals participated in the survey. Table 1 summarizes their demographic and workplace characteristics.

**Table 1:** Participant Characteristics (n = 345) Chi-square tests were performed across professional groups for each variable wherever appropriate. Awareness was measured by the 23-item AMR scale. Data collected using Kobo Collect. Calculated using R software [11-13].

Characteristic	Overall (n = 345)	Doctors (n = 179)	Nurses (n = 82)	Pharmacists (n = 45)	Dentists (n = 39)	p-value	Test/Statistic
Gender, n (%)						<0.001	Chi-square (χ²=72.38), df=6
Male	147 (42.6%)	113 (63.1%)	9 (11.0%)	14 (31.1%)	11 (28.2%)		
Female	197 (57.1%)	66 (36.9%)	72 (87.8%)	31 (68.9%)	28 (71.8%)		
Other	1 (0.3%)	0 (0.0%)	1 (1.2%)	0 (0.0%)	0 (0.0%)		
Age group, n (%)						—	
21–30 years	224 (64.9%)	124 (69.3%)	43 (52.4%)	37 (82.2%)	20 (51.3%)		
31–40 years	99 (28.7%)	45 (25.1%)	29 (35.4%)	6 (13.3%)	19 (48.7%)		
41–50 years	15 (4.3%)	7 (3.9%)	7 (8.5%)	1 (2.2%)	0 (0.0%)		
>50 years	7 (2.0%)	3 (1.7%)	3 (3.7%)	1 (2.2%)	0 (0.0%)		
Level of training, n (%)						—	
University	212 (61.4%)	100 (55.9%)	58 (70.7%)	35 (77.8%)	19 (48.7%)		
Non-university	24 (7.0%)	3 (1.7%)	18 (22.0%)	3 (6.7%)	0 (0.0%)		
Postgraduate	81 (23.5%)	53 (29.6%)	5 (6.1%)	7 (15.6%)	16 (41.0%)		
Specialist	28 (8.1%)	23 (12.8%)	1 (1.2%)	0 (0.0%)	4 (10.3%)		
Type of care, n (%)						<0.001	Chi-square (χ²=36.28), df=6
Primary	163 (47.2%)	65 (36.3%)	40 (48.8%)	32 (71.1%)	26 (66.7%)		
Secondary	62 (18.0%)	32 (17.9%)	18 (22.0%)	2 (4.4%)	10 (25.6%)		
Tertiary	120 (34.8%)	82 (45.8%)	24 (29.3%)	11 (24.4%)	3 (7.7%)		
Type of practice, n (%)						0.076	Chi-square (χ²=11.44), df=6
Private	215 (62.3%)	108 (60.3%)	49 (59.8%)	29 (64.4%)	29 (74.4%)		
Public	73 (21.2%)	34 (19.0%)	21 (25.6%)	8 (17.8%)	10 (25.6%)		
Both	57 (16.5%)	37 (20.7%)	12 (14.6%)	8 (17.8%)	0 (0.0%)		
Attended ABR training, n (%)						0.001	Chi-square (χ²=13.10)
No	264 (76.5%)	146 (81.6%)	59 (72.0%)	25 (55.6%)	34 (87.2%)		
Yes	81 (23.5%)	33 (18.4%)	23 (28.0%)	20 (44.4%)	5 (12.8%)		

Study participants and setting characteristics

This cross-sectional study encompassed 345 healthcare professionals from across Kerala, representing four major healthcare disciplines. The sample comprised 179 physicians (51.9%), 82 nurses (23.8%), 45 pharmacists (13.0%), and 39 dentists (11.3%). 

Educational and practice characteristics varied significantly across professional groups. Among physicians, 55.9% held university degrees, 29.6% had postgraduate training, and 12.8% were specialists. Nurses showed a different pattern, with 70.7% university-trained and 22.0% with non-university qualifications. Pharmacists were predominantly university-educated (77.8%), while dentists were more evenly distributed between university (48.7%) and postgraduate levels (41%).

Practice settings reflected Kerala’s healthcare infrastructure, with 47.2% of participants working in primary care, 34.8% in tertiary care, and 18.0% in secondary care facilities. Private practice predominated (62.3%), followed by public sector employment (21.2%) and mixed practice arrangements (16.5%). This distribution aligns with Kerala’s robust private healthcare sector while maintaining significant public health infrastructure.

AMR awareness scale performance and internal consistency

**Figure 1:**
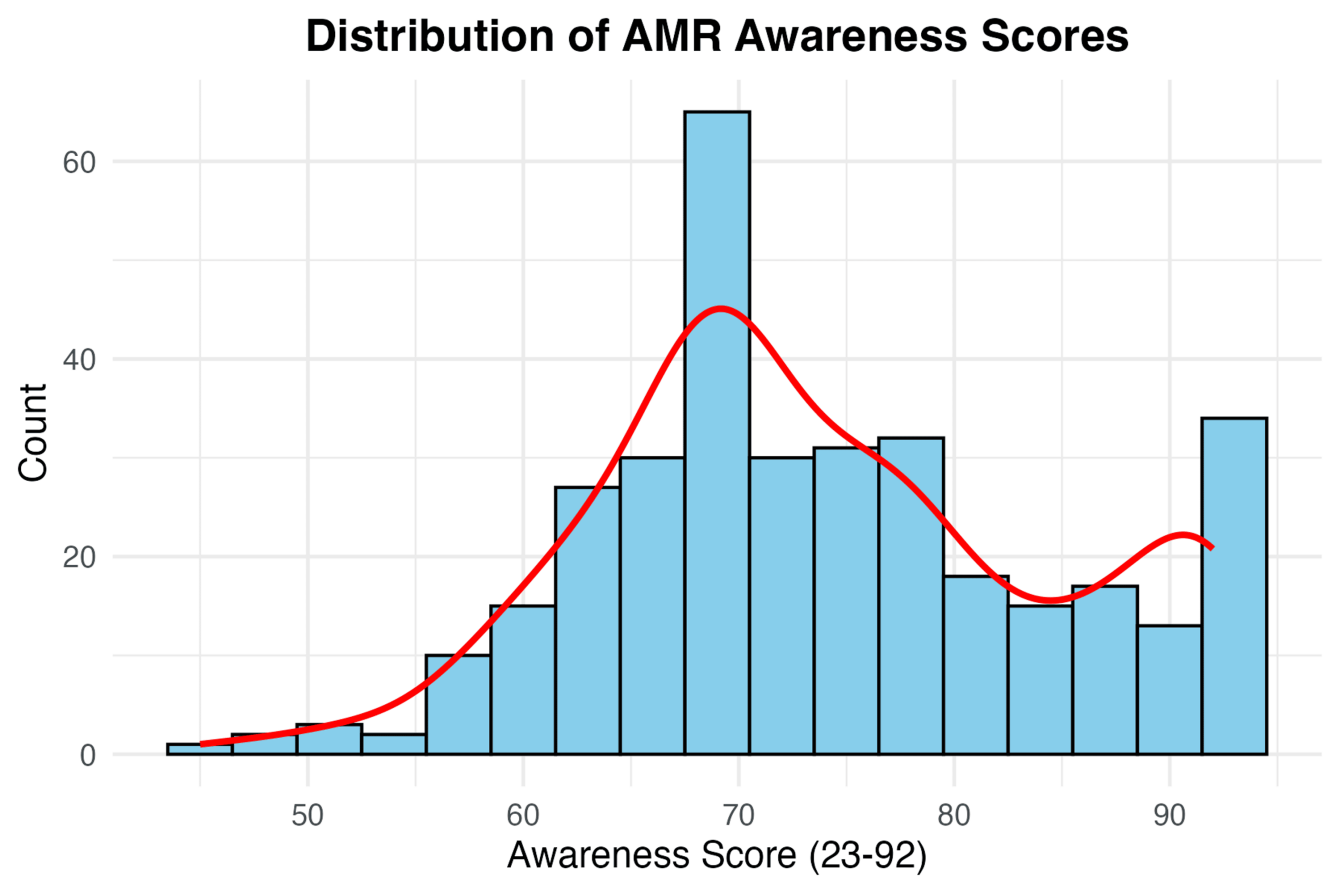
Histogram of total awareness score Awareness was measured by the 23-item AMR scale. Data collected using Kobo Collect. Calculated using R software [11-13].

The validated 23-item AMR Awareness Scale demonstrated excellent psychometric properties with a Cronbach’s α of 0.92, indicating outstanding internal consistency. Overall mean awareness scores reached 73.93 ± 10.50 (range: 45-92), with scores distributed approximately normally across the study population. See Figure 1 for the distribution of AMR awareness scores.

Inter-professional differences in AMR awareness

**Figure 2:**
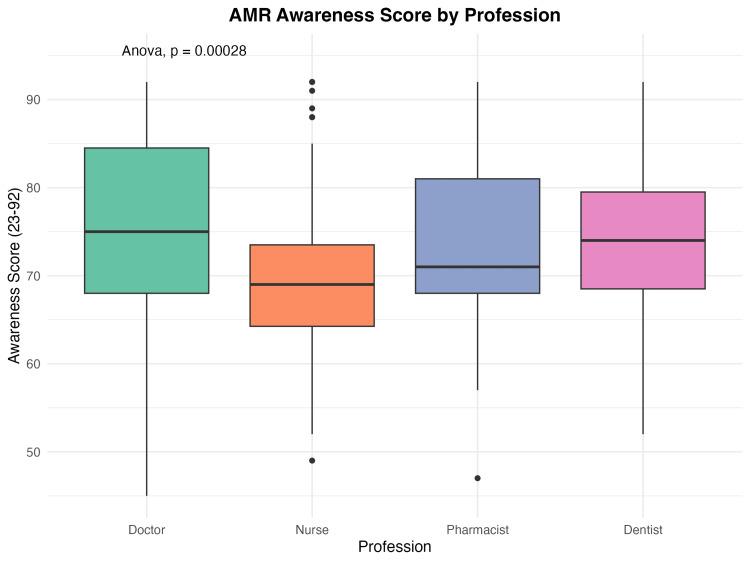
Boxplot of awareness score by profession Awareness was measured by the 23-item AMR scale. Data collected using Kobo Collect. Calculated using R software [11-13].

Significant differences emerged between healthcare professions. Physicians achieved the highest mean awareness scores (75.77 ± 10.31), followed closely by dentists (74.21 ± 10.47) and pharmacists (74.02 ± 10.95), while nurses scored significantly lower (69.73 ± 9.66). See Figure 2 for the awareness score by profession. 

Item-level analysis of AMR awareness components

Consensus was highest for fundamental AMR concepts, with over 94% agreement on core principles: antimicrobial resistance mechanisms (C1: 99.1% agreement), bacterial mutation and resistance development (C2: 98.6%), and infection control measures (C3: 94.8%). Global health threat recognition was also well-established (C23: 94.8%).

**Figure 3:**
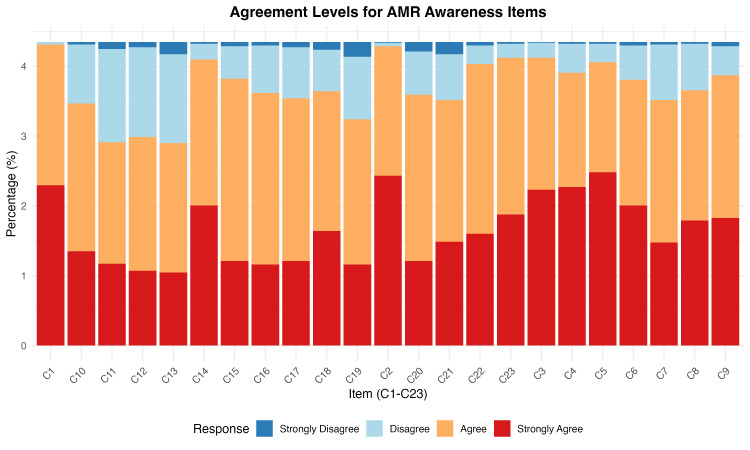
Heatmap of item-level % agreement Awareness was measured by the 23-item AMR scale. Data collected using Kobo Collect. Calculated using R software [11-13].

Knowledge gaps were most pronounced in areas requiring a deeper understanding of resistance mechanisms and clinical applications. Items with lower agreement rates typically involved complex concepts around surveillance, diagnostic stewardship, and antimicrobial optimization strategies. See Figure 3 for agreement levels for AMR awareness items.

Training effects and educational gaps

Formal AMR training attendance was limited across all professions, with only 81 participants (23.5%) reporting specific antimicrobial resistance education. Pharmacists showed the highest training uptake (44.4%), followed by nurses (28.0%), physicians (18.4%), and dentists (12.8%). 

Demographic and contextual associations

Gender differences emerged as significant predictors, with male healthcare professionals scoring higher than females (mean difference: +2.29, p = 0.046). This finding requires careful interpretation, given that the gender distribution varies substantially across professions, with nursing being predominantly female (87.8%) while medicine shows male predominance (63.1%).

Practice setting influenced awareness levels, with tertiary care workers achieving significantly higher scores than primary care colleagues (mean difference: +3.39, p = 0.008). Age showed minimal correlation with awareness (r = 0.041, p = 0.448).

## Discussion

The findings from healthcare workers in Kerala show strengths and serious gaps in their knowledge about antibiotic resistance. These patterns are seen worldwide but also bring out challenges unique to India [6]. The overall mean awareness score of 73.93 shows that the study group had a moderate to high level of knowledge regarding AMR. However, clear differences between professions and education levels point to the need for focused training programs.

The significant physician-nurse awareness gap (mean difference: 6.04 points, p<0.001) is a considerable concern, since nurses are the main administrators of antibiotics and also guide patients for medication. This gap is similar to results from other Indian states and from international studies, showing that nursing education on antibiotic resistance has not received enough attention [6]. Malaysian studies report comparable physician-nurse gaps, while European research highlights that targeted nursing education programs can effectively close these discrepancies. The relatively high pharmacist awareness levels (74.02 ± 10.95) integrate with their specialized pharmaceutical training but still suggest opportunities for improvement in the rational use of antimicrobials [14].

In the present study, only 23.5% of participants reported having received specific training on antimicrobial resistance, with the highest uptake observed among pharmacists (44.4%), followed by nurses (28.0%), physicians (18.4%), and dentists (12.8%). These findings are consistent with those of a previous study, in which only 18% of physicians reported attending short-term Continuing Medical Education (CME) courses on the judicious use of antibiotics, and none had undergone formal antimicrobial stewardship training. Both studies highlight a persistent gap in formal AMR training, particularly among physicians, who play a central role in antibiotic prescribing. Additionally, our results extend the existing evidence by demonstrating notable differences across professional groups, with pharmacists and nurses showing relatively higher engagement in AMR-related education compared with physicians and dentists [15].

The comparable awareness scores among doctors, pharmacists, and dentists indicate that health science education at the university level gives a basic understanding of antimicrobial resistance, but applying this knowledge in specific professions needs focused training. This finding supports calls for interprofessional AMR education that utilizes shared knowledge bases while addressing discipline-specific competencies [14]. 

There is a clear inverse relationship between initial awareness and participation in training, which poses a challenge for expanding AMR education. Doctors, despite having the highest awareness scores, attended training the least (18.4%), whereas pharmacists with high awareness had the highest participation (44.4%). This indicates that the current training programs may not be reaching the right audience effectively.

The non-significant but positive training effect (2.39-point improvement, p = 0.074) corresponds with evidence that AMR educational interventions have modest but valuable impacts. A recent systematic review of 19 studies found similar effect sizes, with clinical significance often preceding statistical significance in educational interventions targeting complex knowledge domains [6].

Kerala's well-developed healthcare system offers a strong foundation for expanding effective training programs, but the overall training participation of 23.5% indicates that many opportunities are being missed. Successful programs in Maharashtra and Karnataka achieved 60-70% participation rates through mandatory continuing education integration and professional licensing requirements [16].

The male-female awareness gap requires careful interpretation. The gender distribution across professions varies, and there might be possible differences in access to education. Our sample shows female predominance (87.8%) in nursing. Although the difference in mean awareness scores between male and female participants was statistically significant, the limited magnitude of this difference indicates limited practical significance. Therefore, this gender difference is unlikely to have a meaningful clinical or educational impact compared to the larger disparities observed across healthcare professions and training exposure.

In the present study, practice setting was found to influence awareness levels significantly, with tertiary care workers achieving higher scores than their primary care counterparts (mean difference: +3.39, p = 0.008). This finding is consistent with prior research, where knowledge levels varied according to professional hierarchy and training background. For instance, one study reported that faculty members demonstrated the highest overall knowledge (90.7%), followed by residents (84%) and interns (72.4%) [17]. However, this gradient poses challenges for Kerala's primary care-focused health system, where most antibiotic prescribing occurs. Effective bridging strategies in other Indian states have used telemedicine consultation and mobile educational units to extend tertiary expertise to primary settings [16].

The Kerala Antimicrobial Resistance Strategic Action Plan, KARSAP, was the first state-level AMR action plan launched in India. The state should view this as an opportunity to implement a robust, periodic training strategy for the healthcare workforce. These findings can be utilized to enhance resource distribution and focus interventions effectively. Immediate efforts should be made to reduce the awareness gap between doctors and nurses by organizing joint training programs that leverage physicians' higher baseline knowledge [8]. Karnataka's successful nurse-led AMR programs demonstrated 15-20% improvement in awareness scores through peer-to-peer learning models [16].

There is a critical need for targeted and sustained antimicrobial stewardship training among healthcare professionals. Profession-specific educational interventions, particularly focusing on nurses and primary care providers, are essential to build capacity, improve rational antibiotic use, and enhance stewardship program effectiveness in Kerala’s healthcare system. Tamil Nadu's integration of AMR competencies into the medical council licensing achieved 85% training participation within two years [16]. Medium-term strategies should tackle the low training participation by introducing professional development incentives and linking advancement to competency-based requirements. Government-led initiatives focusing on policy, funding, regulation, and education can successfully bring about positive changes in the healthcare workforce, improve antibiotic use, and safeguard public health [8]. 

Limitations 

While our sample intentionally included diverse healthcare professions consistent with Kerala’s interprofessional stewardship strategy and aligned with the validated 23-item scale by Pinto Jimenez et al., we recognize the heterogeneity limits generalizability. Therefore, the results should be interpreted within the context of the specific professional groups included in the study [8,11]. It is important to note that doctors, primarily from the private sector, constituted the largest participant group in this study. This reflects the significant role of private healthcare providers in Kerala but may limit the generalizability of the findings to other healthcare worker groups and public sector providers [18].

The convenience sampling method involved sharing the online survey link through various open-access channels to target a diverse group of healthcare professionals. However, this approach prevented us from calculating an accurate response rate. Selection bias toward more active healthcare professionals may limit how well the findings apply to Kerala’s wider healthcare workforce [14]. Given the self-selected nature of participation via online and network-based invitations, the study may also be subject to non-response bias if individuals who are more interested or knowledgeable about AMR were disproportionately represented. This factor, combined with the convenience sampling approach, could have led to a slight overestimation of awareness within certain professional groups. Our study did not gather information regarding the frequency, duration, or recency of AMR training. Future studies should address this to better understand the impact of repeated or ongoing educational interventions.

Recommendations

Research priorities to handle antimicrobial resistance include testing specific training with randomized trials, measuring long-term results, and examining how to effectively add these programs into health systems. The validated awareness scale serves as a useful tool for these studies. Given the findings on interprofessional awareness gaps, future studies with expanded sample sizes per profession are essential to strengthen subgroup analyses and develop more tailored interventions.

With its high literacy, solid healthcare infrastructure, and government support, Kerala is an ideal place to test these interventions. Successful models here could guide nationwide adoption under India’s National Health Mission. Also, similar large-scale multi-state replication studies are vital for national policy development [7,8].

## Conclusions

This study shows that while Kerala’s healthcare workforce has a solid basic understanding of antimicrobial resistance, gaps remain between different professional groups and care settings. Differences between physicians and nurses, limited training participation, and disparities between primary and tertiary care need to be addressed to strengthen antimicrobial stewardship. Focusing on nursing education, mandatory professional training, and support for primary care can make the most of Kerala’s healthcare infrastructure. With continued political commitment, adequate resources, and systematic evaluation, Kerala has the potential to serve as a model for AMR education and control, offering lessons that could guide broader efforts across India and beyond.

## Appendices

Questionnaire

Awareness of Antibiotic Resistance: A Tool for Measurement Amongst Health Care Professionals

Antibiotic resistance awareness among healthcare professionals was assessed using the validated 23-item scale developed by Pinto Jimenez et al. (2023). This instrument is free to use for non-commercial research under a Creative Commons Attribution-Noncommercial License (CC BY-NC 4.0). As per the license, non-commercial re-use, distribution, and reproduction in any medium are permitted, provided the original work is properly cited.

**Table 2:** Antibiotic resistance awareness among healthcare professionals tool Item A1 – A7: Demographics module, Item B1 – B3: ABR training module, Item C1 – C24: Antibiotic resistance awareness module (Health Care Professional Antibiotic Resistance Awareness Scale v1), Item D1.1 - D3.11: Context module (D1.1 - D1.6: Prioritisation of AMR, D2.1 - D2.8: Resources available, D3.1 - D3.11: Information about AMR) [11].

Item ID	Questions / items	Variable description
A1	Country	
A2	Profession	Physician, Nurse, Dentist, Physician assistant, Medical officer, Pharmacist
A3	Gender	Male, Female
A4	Age	Continuous, recoded as 18 to 25, 26 to 35,36 to 45, 46 to 55 or > 55
A5	Level of Training	Non-university, University, Specialist, Postgraduate
A6	Type of care	Primary, secondary, tertiary
A7	Type of practice	Public, Private or Both
B1	I was taught everything I needed to know about Antibiotic Resistance as part of my training curriculum	Likert
B2	The information and training I currently receive on Antibiotic Resistance are adequate for my day-to-day practice	Likert
B3	I have attended specific training on Antibiotic Resistance and/or Antibiotic Stewardship	Yes/No
C1.	Antibiotic resistance is when a microorganism becomes resistant to antibiotics	Likert
C2.	Some microorganisms can mutate and therefore become resistant to antibiotics	Likert
C3.	Some microorganisms can transfer resistance by exchanging genetic material	Likert
C4.	Antibiotic resistance can develop if antibiotics are given when they are not indicated, for example, when a person has a viral infection	Likert
C5.	Antibiotic resistance can develop if courses of antibiotic treatment are interrupted, for example, stopping and starting again halfway through a prescribed course	Likert
C6.	Antibiotic resistance can develop if antibiotics are given/taken in lower than recommended doses	Likert
C7.	Antibiotic resistance can develop if antibiotics are used to treat bacterial colonisation rather than bacterial infection	Likert
C8.	Antibiotic resistance can develop if antibiotics are used as a ‘just in case measure’ for any routine procedure	Likert
C9.	Antibiotic resistance can develop if broad-spectrum antibiotics are used when a narrow-spectrum antibiotic would resolve the infection	Likert
C10.	Antibiotic resistance can develop if antibiotics are used in livestock feed to promote animal growth	Likert
C11.	Antibiotic resistance can develop if human antibiotics are used to treat infections in animals	Likert
C12.	Antibiotic resistance can develop if antibiotics are present in human sewerage	Likert
C13.	Antibiotic resistance can develop if antibiotics are discarded into the environment	Likert
C14.	Resistant infections can spread from health care facilities including hospitals	Likert
C15.	Resistant infections can spread within residential areas	Likert
C16.	Resistant infections can spread from livestock farms	Likert
C17.	Resistant infections can spread through wastewater	Likert
C18.	Strict hand hygiene before and after contact with patients can help prevent the spread of antibiotic resistance between patients	Likert
C19.	Isolation in a single room can help prevent the spread of antibiotic resistance between patients	Likert
C20.	Appropriate environmental cleaning can help prevent the spread of antibiotic resistance between patients	Likert
C21.	Wearing personal protective equipment such as gloves, masks and aprons can help prevent the spread of antibiotic resistance between patients	Likert
C22.	I recognise that a person has a resistant infection when the person remains unresponsive to a number of different antibiotics	Likert
C23.	I recognise that a person has a resistant infection by sending them for culture and sensitivity testing at a laboratory	Likert
D1.1	If I do not prescribe/dispense an antibiotic, there could be a worse health outcome for the person I am treating	Likert
D1.2	At my place of work, I consider malnutrition a higher concern than Antibiotic Resistance	Likert
D1.3	At my place of work, I consider chronic disease a higher concern than Antibiotic Resistance	Likert
D1.4	At my place of work, I consider the level of hygiene and sanitation a higher concern than Antibiotic Resistance	Likert
D1.5	At my place of work, I consider other infectious diseases (TB/Malaria/HIV) a higher concern than antibiotic resistance	Likert
D1.6	At my place of work, I consider trauma and accidents (for example, traffic accidents and burns) a higher concern than antibiotic resistance	Likert
D2.1	The availability of laboratory services affects my decision to prescribe/dispense antibiotics	Likert
D2.2	My facility has the capacity to provide culture and sensitivity testing	Yes/No
D2.3	There is a nearby facility that I can send samples to if I need culture and sensitivity testing	Yes/No
D2.4	I am confident that the facility I use for culture and sensitivity testing has functional equipment	Yes/No
D2.5	I am confident that the facility I use for culture and sensitivity will always be fully staffed	Yes/No
D2.6	I always have running water at my place of work	Yes/No
D2.7	I always have electricity at my place of work	Yes/No
D2.8	I am always able to prescribe/dispense the best antibiotic for the infection I am treating at my place of work	Yes/No
D3.1	I have access to data on local Antibiotic Resistance patterns	Yes/No
D3.2	I receive data on local Antibiotic Resistance patterns at my place of work	Yes/No
D3.3	Someone at my place of work is monitoring Antibiotic Resistance	Yes/No
D3.4	I am exposed to advertising on antibiotics	Yes/No
D3.5	I am aware of campaigns about antibiotic resistance	Yes/No
D3.6	Medical representatives provide health care workers with information on antibiotics	Never/Often/Sometimes
D3.7	Medical representatives provide health care workers with samples of antibiotics	Never/Often/Sometimes
D3.8	I would like to receive more information about Antibiotic Resistance	Yes/No
D3.9	I would like to receive more information about Antibiotic Resistance at work	Yes/No
D3.10	I would like to receive more information about Antibiotic Resistance online	Yes/No
D3.11	I would like the opportunity to take part in further training regarding Antibiotic Resistance	Yes/No
